# Diagnostic and treatment pathways for men with prostate cancer in Queensland: investigating spatial and demographic inequalities

**DOI:** 10.1186/1471-2407-10-452

**Published:** 2010-08-23

**Authors:** Peter D Baade, Joanne F Aitken, Megan Ferguson, Robert A Gardiner, Suzanne K Chambers

**Affiliations:** 1 Viertel Centre for Research in Cancer Control, Cancer Council Queensland, PO Box 201, Spring Hill QLD 4004, Australia; 2 School of Public Health, Queensland University of Technology, Herston Road, Kelvin Grove 4059, Australia; 3 School of Population Health, University of Queensland, Herston Road, Herston 4061, Australia; 4 University of Queensland Centre for Clinical Research, Royal Brisbane Hospital, Herston, 4029, Australia; 5 Department of Urology, Royal Brisbane Hospital, Herston, 4029, Australia; 6 Griffith Health Institute, Griffith University, Brisbane, Australia

## Abstract

**Background:**

Patterns of diagnosis and management for men diagnosed with prostate cancer in Queensland, Australia, have not yet been systematically documented and so assumptions of equity are untested. This longitudinal study investigates the association between prostate cancer diagnostic and treatment outcomes and key area-level characteristics and individual-level demographic, clinical and psychosocial factors.

**Methods/Design:**

A total of 1064 men diagnosed with prostate cancer between February 2005 and July 2007 were recruited through hospital-based urology outpatient clinics and private practices in the centres of Brisbane, Townsville and Mackay (82% of those referred). Additional clinical and diagnostic information for all 6609 men diagnosed with prostate cancer in Queensland during the study period was obtained via the population-based Queensland Cancer Registry.

Respondent data are collected using telephone and self-administered questionnaires at pre-treatment and at 2 months, 6 months, 12 months, 24 months, 36 months, 48 months and 60 months post-treatment. Assessments include demographics, medical history, patterns of care, disease and treatment characteristics together with outcomes associated with prostate cancer, as well as information about quality of life and psychological adjustment. Complementary detailed treatment information is abstracted from participants' medical records held in hospitals and private treatment facilities and collated with health service utilisation data obtained from Medicare Australia. Information about the characteristics of geographical areas is being obtained from data custodians such as the Australian Bureau of Statistics. Geo-coding and spatial technology will be used to calculate road travel distances from patients' residences to treatment centres. Analyses will be conducted using standard statistical methods along with multilevel regression models including individual and area-level components.

**Conclusions:**

Information about the diagnostic and treatment patterns of men diagnosed with prostate cancer is crucial for rational planning and development of health delivery and supportive care services to ensure equitable access to health services, regardless of geographical location and individual characteristics.

This study is a secondary outcome of the randomised controlled trial registered with the Australian New Zealand Clinical Trials Registry (ACTRN12607000233426)

## Background

Internationally, prostate cancer is the second most common cancer diagnosed among men worldwide (12% of all cancers) and the 6th most common cause of cancer-related deaths [1]. Prostate cancer is particularly prevalent in developed countries such as the United States, Scandinavian countries, Canada and Australia, with about a six-fold difference between high-incidence and low-incidence countries[2]. Among Australian males in 2010, prostate cancer is expected to have the second highest cancer-related disease burden, measured by disability-adjusted life years (DALYs), behind lung cancer [3].

Based on autopsy studies, substantial proportions of men have histological prostate cancer that doesn't cause symptoms or death [4,5]. Since the introduction of PSA testing, increasing numbers of these indolent prostate cancers are now being diagnosed [6]. As such, many of the prostate cancers being diagnosed will have little impact on a man's health even in the absence of treatment [7] and that treatment may in fact be harmful as it may carry significant side effects with no potential benefit. However many prostate cancers do have the potential to metastasise and cause death. Worldwide, there were nearly 260,000 deaths caused by prostate cancer in 2008, with 53% of these being in developed regions[8]. We have shown previously that a diagnosis of prostate cancer has greater implications for men diagnosed at a young age, with these men being more likely to die prematurely[9]. Unfortunately it is not currently possible to differentiate the indolent cancers from those that have the potential to metastasise before biopsy, thus making the important decision of whether to be screened and subsequent treatment decisions potentially complex and distressing.

For localised prostate cancer, potentially curative treatment options include surgery through radical prostatectomy and radiation therapy (external beam and brachytherapy). While these surgical or radiation treatments may increase the chance of cure, they are associated with a significant risk of impotence, and less commonly urinary incontinence and bowel problems. The prevalence and type of side-effects vary for different treatment types[10]. Another treatment option is active surveillance, in which treatment is initially deferred, then initiated if the disease progresses. This differs from "watchful waiting" in which medical treatment is reserved until symptoms become evident, thus potentially missing the opportunity for cure. In contrast close observation maintains the likelihood of cure by intervening before symptoms present.

The contrasting initial results recently published from two large-scale prostate cancer screening trials in the United States [11] and Europe [12] highlight the complexity of issues surrounding the provision of diagnostic and treatment services for prostate cancer. In the presence of already high PSA testing, the United States study suggested that organised PSA population screening programs are likely to result in over-diagnosis and over-treatment with little reduction in mortality. Conversely, in Europe where the prevalence of asymptomatic detection is low, there is greater potential for screening programs to reduce the number of advanced prostate cancers and deaths from prostate cancer. A sub-study conducted in Göteborg, Sweden, a country with a low prevalence of PSA testing (3%)[13] found that using an intention to treat analysis PSA testing almost halved the risk of death from prostate cancer. A major difference between this study and its two larger international predecessors is its more appropriate duration of follow-up viz. 14 years.

Internationally, there is substantial evidence that socio-demographic factors, such as age, ethnicity, location of residence and income are associated with prostate cancer outcomes extending from the likelihood of a man having a PSA test to differences in prostate cancer management [2,14-18]. In Australia [15] there has been a statistically significant and increasing excess of prostate cancer mortality among men in regional and rural areas compared with those living in capital cities. This mortality excess was accompanied by lower rates of radical prostatectomy and PSA testing in rural and regional Australia, and was in spite of lower incidence rates of prostate cancer.

At present, the patterns of care for men in Queensland, Australia, are not documented in any systematic way. For example, for men diagnosed with prostate cancer, we currently do not know which medical treatments they receive, what factors are associated with different choices, nor the morbidity and mortality risks associated with these different treatments in their settings. Since this information is needed for the planning and development of health delivery and supportive care services and to ensure equitable access to health services, this longitudinal study of men diagnosed with prostate cancer in Queensland was undertaken.

### Study Aims

Utilising a longitudinal study design and recruiting men immediately after their initial diagnosis, this study aims to obtain information about their initial symptoms and diagnosis, and then prospectively follow them during subsequent management to identify their pathways to care and subsequent outcomes. Specifically it aims to:

1. Document the current diagnostic and treatment patterns for men with prostate cancer, in Queensland, Australia;

2. Investigate the determinants of area-level and individual-level variation in diagnostic and treatment pathways;

3. Investigate the determinants of area-level and individual-level variation in clinical, psychosocial and survival outcomes following treatment for prostate cancer;

4. Identify inequalities in diagnostic and treatment pathways, with a view to translating that information into health policy and practice decision-making.

## Methods/Design

### Study Design

This is a descriptive, longitudinal epidemiological study, in which is embedded a randomised controlled trial of a decision support intervention for men with localised prostate cancer. The descriptive epidemiology component is a secondary outcome of the randomised controlled trial [19] and has been registered with the Australian New Zealand Clinical Trials Registry (registration number ACTRN12607000233426).

### Funding and Support

This project was awarded funding by the (Australia) National Health and Medical Research Council (ID: 442301) and a Cancer Council Queensland Research Grant. Cancer Council Queensland provided additional funding for the maintenance of the GIS software.

### Ethical Clearance

The full study, including documentation of the patterns of diagnosis and treatment, has been approved by the Queensland University of Technology Human Research Ethics Committee, in addition to the ethics committees of ten public hospitals in Queensland. The collection of Medicare data has been approved by Medicare Australia and access to data from the Queensland Cancer Registry was approved by Queensland Health.

### Setting

Men diagnosed with prostate cancer in Queensland (Australia) between February 2005 and July 2007 (inclusive) are eligible for this study. As the second largest area of the Australian states and territories, Queensland has the third highest population of any state (4.4 million in 2009) and is the most decentralised [20]. Recruitment is centred on the two major tertiary treatment centres for prostate cancer in Queensland; the greater Brisbane and Townsville areas.

### Study areas

Participants were ascertained through urology outpatient clinics in the Greenslopes Private, Royal Brisbane, Mater Adults, Princess Alexandra, Ipswich, QEII, Redlands, and Redcliffe Hospitals in Brisbane (and surrounds) and through the Townsville General Hospital and Mackay Base Hospital. Private patients were ascertained through the private practices of Urologists in Brisbane (and surrounds), Townsville and Mackay.

### Participants

#### Study cohort

All men newly diagnosed with prostate cancer in Queensland between 1st February 2005 and 31st July 2007 were potentially eligible for the study. Urologists referred eligible men to the study if they were newly diagnosed with prostate cancer irrespective of stage of disease. The following exclusion criteria were used: previous diagnosis of prostate cancer; inability to read, write and speak English; history of dementia, head injury or psychiatric illness; and no regular access to a telephone.

**Figure 1:**
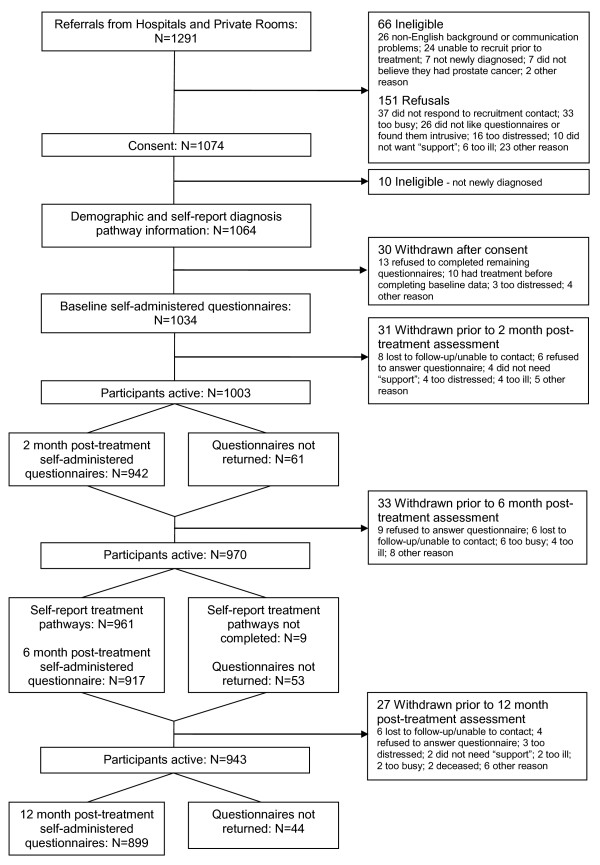
**Flowchart of recruitment, participation, data collection and attrition from study**.

In all, 1291 men were referred to the study, with 1064 men (82.4% of referrals) eligible and agreeing to participate. Figure 1 shows the flowchart of ineligible/excluded men, completeness of data collection and attrition from the study between recruitment and 12 months post-treatment.

#### Population cohort

Clinical and diagnostic information for *all *men diagnosed with prostate cancer in Queensland during the study period (February 2005 to July 2007), whether or not they are in the study cohort, was accessed via the Queensland Cancer Registry. This population-based data will enable a comparison of demographic, geographical, clinical and diagnostic information between the study cohort and men diagnosed with prostate cancer who did not take part in the study (n = 5545).

### Individual-level data

#### Telephone and self-administered questionnaires

Data collection for the study cohort is via telephone and self-administered questionnaires and occurs at pre-treatment and 2 months, 6 months, 12 months, 24 months, 36 months, 48 months and 60 months post-treatment. These assessments include demographic information, medical history, self-reported diagnostic and treatment information together with outcomes associated with prostate cancer, including information about quality of life and psychological adjustment. The quality of life and psychological measures used in this study are well-established and validated and have been described previously[19].

##### Demographics

the demographic information collected at baseline includes date of birth, marital status, ethnicity, education level, income, current employment status and health insurance.

##### Medical history

includes family history of cancer, family history of prostate cancer, body mass index (BMI), other chronic conditions and co-morbidities and smoking status.

##### Self-reported diagnostic and treatment information

This self-reported information includes PSA level, Gleason score, previous screening history, the diagnostic examinations performed together with the date and location of each examination, the reasons leading to the initial diagnosis, reasons for delaying the diagnosis, the type of treatment undertaken and the date and location of each type of treatment and the public or private status of the treating facility. Detailed self-report diagnostic and treatment information is collected on all participants up to 6 months after primary treatment commenced. We will continue to collect basic information on disease progression and subsequent treatments up to 60 months following primary treatment.

#### Medical Records

For the study cohort, detailed treatment information is being abstracted from participants' medical records held in hospitals and private treatment facilities. This includes details of diagnosis and post-treatment PSA levels, TNM Staging, diagnostic examinations performed and the dates and locations of these examinations, the types of treatment and the treating doctors and their facilities. Any information pertaining to further treatments such as radiation therapy, androgen deprivation therapy and other medications is being collected as relevant. Detailed information from medical records is collected from diagnosis to 12 months after primary treatment was started.

#### Medicare Information

Study participants are being asked to sign an additional consent form to facilitate access to their medical records through Medicare Australia. Data relating to health service utilisation including general practitioner and specialist services, prescription drug use (from the Pharmaceutical Benefits Scheme), diagnostic and imaging tests and procedures is being obtained. Information requested from Medicare Australia will cover a 7 year period starting from the 1^st ^January 2005 for each study participant.

#### Queensland Cancer Registry data collection

For the entire population cohort, information is obtained from the Queensland Cancer Registry (QCR) regarding the clinical details recorded for each prostate cancer diagnosis. These details include the Gleason score, the tumour volume, number of positive cores, the extent of invasion (including extraprostatic extension, seminal vesicle invasion and vascular invasion), and, when radical prostatectomy is performed and a lymhadenectomy is also undertaken, the number of positive lymph nodes. When available and relevant, details are obtained from multiple pathology reports sourced within the Queensland Cancer Registry including transrectal ultrasound (TRUS), Transurethral Resection of the Prostate (TURP), prostatectomy, lymph node dissection and bone biopsy pathology reports.

Information will also be gathered from the QCR on date of diagnosis, place of usual residence at diagnosis, age group at diagnosis, gender, country of birth, marital status, occupation and Indigenous status. Although there is evidence of some under-identification, the Indigenous identifier in Queensland is considered to have good coverage [21].

### Area-level information

The smallest geographical entity used for this analysis is Collection Districts (CDs) which cover Queensland without gaps or overlap. There were 7567 populated CDs in Queensland in 2006, with a median population of 497 (range: 3 to 2372). These CDs can then be collapsed to form Statistical Local Areas (SLA) using a deterministic (m:1) match between the CDs and the SLAs.

These SLAs are often based on local government administrative areas, with the local governments being responsible for local and regional service provision and infrastructure. In 2006 there were 478 SLAs in Queensland with a median population of 5810 (range: 7 to 77523). The SLA is also used as the standard geographical area definition by most relevant data providers, in particular the Queensland Cancer Registry and Australian Bureau of Statistics. The use of geo-coding enables us to match all area-level information to the 2006 Australian Standard Geographic Classification definition, thus removing any impact of changes in these geographic boundaries over time.

#### Remoteness

Remoteness of residence when diagnosed with breast cancer was categorized using the ARIA+ classification [22], which categorizes remoteness into Major City, Inner Regional, Outer Regional, Remote and Very Remote areas. The ARIA+ classification is a purely geographic measure that considers road distance measurements from localities, and is a measure of accessibility and remoteness.

#### Socioeconomic status

Area-level socioeconomic disadvantage is measured using the Index of Relative Socioeconomic Disadvantage (IRSD) calculated by the Australian Bureau of Statistics [23]. The IRSD provides a general measure of disadvantage, and considers factors such as low income, low educational attainment, high unemployment, jobs in relatively unskilled occupations.

#### Census data

The Australian Bureau of Statistics conducts a population Census every 5 years, with the latest available data currently available for 2006. Information is available down to CD level, and then can be aggregated up to SLA level. Variables include occupation, household income, number of vehicles per occupied private dwelling, marital status, Indigenous status, labour force status and highest educational qualification.

Details about occupation were collapsed into "Blue Collar" (including tradespersons, plant and machine operators and drivers, and labourers and related workers), "White collar" (including clerks, salespersons and personal service workers), "Professional" (including managers and administrators, professionals and para-professional), "Not in workforce" (including retired, students, unemployed and home duties), "Unknown" (no information available). Similar occupation categories have been used previously [24].

##### Geocoding of street address information

All address details for study respondents, diagnosing and treating doctors and treatment facilities are geo-coded using commercial software designed to clean and convert street address information into spatial (latitude and longitude) coordinates. When full street address information for a patient, doctor or treating facilities is not available, the centroid of the SLA will be used instead.

##### Road travel distance calculations

Distances between patients, diagnostic centres, treatment facilities and other medical centres will be calculated directly from the geo-coded latitude and longitude points. Many studies assessing distance to treatment have calculated straight line distances [25-31], however this method may under-estimate travel times [29], illustrating the importance of using accurate road distance and travel time information [25,27]. These road distances and times will be calculated using commercial GIS software, combined with street network analysis and display tools: and custom GIS applications to enable calculation of the closest road travel distance between one location (such as patient's residence) and multiple locations (such as various treatment facilities).

##### Primary Outcome Measures

Diagnostic pathway: a description of events and time line from first noticing symptoms or presenting to a medical practitioner to the definitive diagnosis. This includes the diagnostic interval, defined as the time between the initial doctor's consultation for reasons that led to the prostate cancer diagnosis, and the date of definitive diagnosis.

Treatment pathway: a description of events and time line from the definitive diagnosis to 6 months post the commencement of primary treatment. This includes the treatment interval, which is the time between the definitive diagnosis and the commencement of primary treatment for the prostate cancer.

Treatment details: a description of the type, duration and frequency of all treatments received by the participant between diagnosis and 6 months after the commencement of primary treatment. Primary treatment is considered the first most invasive treatment undergone by the participant and is generally radical prostatectomy or radiation therapy (either external beam radiotherapy or brachytherapy). For participants who chose watchful waiting or active surveillance, date of treatment is considered as the date the treatment decision is made.

### Statistical analysis

Statistical analyses for this study will reflect the sampling design, the data collection process, and the research objectives as stated previously. Due to the typically highly skewed distributions of time to diagnosis or treatment, standard methods for transforming the time intervals such as the geometric mean or comparing median times, will be used in the analyses. Crude and adjusted logistic and multinomial regression analyses of dichotomous and ordinal outcomes respectively, will be used to assess the contribution that independent factors have on men's choice of treatment. We will also investigate the representativeness of the study cohort compared to the rest of the men diagnosed with prostate cancer by comparing the distributions of those clinical and demographic variables collected from the Queensland Cancer Registry.

Previous studies looking at patterns of diagnosis and treatment have focussed on the individual-level data, or ecological studies. In order to appropriately investigate whether the observed patterns are due to the characteristics of the cancer patients themselves, or the characteristics of the geographical areas in which the individuals live, it is important to be able to quantify the variation between areas in terms of their diagnostic and treatment profiles. Thus using a multilevel analytical design we can then determine whether the observed variation is due to the clustering of individuals (i.e. a composition effect) or the environmental characteristics of the areas (i.e. a context effect).

We have previously described the advantages of incorporating a multilevel perspective [32]. We will use multilevel models to assess whether geographic remoteness and area-level disadvantage were associated with prostate cancer outcomes after controlling for individual-level socio-demographic characteristics. These models will be fitted using MLwiN version 2.15 (University of Bristol, United Kingdom).

There will be three stages in the model building process. The first is the null model that including individuals (level 1) nested in SLAs (level 2), there are no individual- or area-level variables in the fixed part of this model. If there is a significant SLA-level random term (indicated using Wald chi-square) then this is evidence supporting significant between-SLA variation in the prostate cancer outcome. Second, the null model will be extended by including individual-level factors (patient characteristics, disease stage, co-morbidity, and access to health care and treatment services) as fixed effects. This will inform us as to how much of the area-variation in prostate cancer outcomes is due to these individual-level (compositional) factors, and also assess the contribution of each individual-level factor. Thirdly, the area-level measures such as geographic remoteness and area socioeconomic disadvantage will be included in the model as fixed effects, to quantify how much of the area-variation in prostate cancer outcomes is due to these factors (independent of individual-level factors). Subsequent models will also consider the cross-level interactions between the individual-level and area-level factors.

#### Power calculations

Cohort sample sizes were initially determined in order to calculate differences in binomial proportions between two equally-sized subgroups. Assuming pooled variance, two-sided Z-test at 5% significance level, subgroup sample sizes of n = 500 men achieve 80% power to detect at least an absolute difference between the subgroup proportions of 9%.

Power calculations for the variation between and within area units (using *Optimum Design *software[33]) are based solely on the approximate number of clusters (statistical local areas) and records per cluster. The study cohort covers 270 area units (SLAs), with between 1 and 66 (mean 3.9, median = 2) respondents in each unit. Assuming 270 clusters with an average of 4 records per cluster and a baseline proportion of 50%, this gives 80% power at 0.05% significance to detect a difference in proportions between two subgroups of 11%.

## Discussion

Prostate cancer is a cause of significant morbidity and mortality among Western populations, including Australia, and carries with it substantial individual and public health burden. Currently, little is known about the diagnostic and treatment patterns of men diagnosed with prostate cancer in this setting and the extent to which men who are diagnosed with this cancer have equitable access to different treatment options and follow up care. This lack of knowledge means that health services policy and planning strategies to manage this illness are to a large extent unguided by evidence. The present study will address this knowledge and evidence gap by investigating the diagnostic and treatment patterns of men diagnosed with prostate cancer in Queensland; assessing the extent to which these patterns systematically vary; and the area level and individual factors that contribute to any variation. We propose that this will provide a basis for advocacy for improvements in prostate cancer care and men's health in general.

## Competing interests

The authors declare that they have no competing interests

## Authors' contributions

PB led the writing for this manuscript. SC, JA and FG conceived the initial study and SC led the grant applications for funding for this study. All authors have contributed to the design and data collection protocols. All authors have read and approved the manuscript.

## Pre-publication history

The pre-publication history for this paper can be accessed here:

http://www.biomedcentral.com/1471-2407/10/452/prepub
